# Pharmacokinetic/pharmacodynamic analysis of sulbactam against *Acinetobacter baumannii* pneumonia: establishing *in vivo* efficacy targets in the epithelial lining fluid

**DOI:** 10.1093/jacamr/dlae203

**Published:** 2024-12-20

**Authors:** Yasmeen Abouelhassan, Joseph L Kuti, David P Nicolau, Kamilia Abdelraouf

**Affiliations:** Center for Anti-Infective Research and Development, Hartford Hospital, Hartford, CT, USA; Center for Anti-Infective Research and Development, Hartford Hospital, Hartford, CT, USA; Center for Anti-Infective Research and Development, Hartford Hospital, Hartford, CT, USA; Division of Infectious Diseases, Hartford Hospital, Hartford, CT, USA; Center for Anti-Infective Research and Development, Hartford Hospital, Hartford, CT, USA

## Abstract

**Background:**

Sulbactam is an effective therapy for *Acinetobacter baumannii* infections. Previous sulbactam pharmacokinetics/pharmacodynamics (PK/PD) analyses established exposure efficacy targets in plasma against *A. baumannii* pneumonia. Herein, we established sulbactam efficacy targets in epithelial lining fluid (ELF). The PTA following clinical sulbactam regimens was estimated.

**Methods:**

Sulbactam (dosed as ampicillin-sulbactam) bronchopulmonary PK was assessed in the neutropenic murine pneumonia model. The percentage of the dosing interval during which the free drug concentration remained above the MIC (%*f*T > MIC) required to achieve different efficacy endpoints was estimated in 21 clinical *A. baumannii* isolates. PTA was assessed using Monte Carlo Simulations and utilizing previously published healthy volunteers sulbactam ELF pharmacokinetics.

**Results:**

Median (IQR) %*f*T > MIC required to achieve 1-log kill in isolates resistant to both sulbactam and meropenem was 47.51 (39.7–54.2). This target was much higher than isolates with other phenotypes (i.e. sulbactam-susceptible/intermediate and sulbactam-resistant but meropenem susceptible) that required 16.62 (5.3–22.0). The PTA following sulbactam 1 g q6h 0.5h infusion regimen was >90% up to MIC of 2 mg/L while the PTA for MIC 4 mg/L (susceptibility breakpoint) was 81%. Conversely, previous assessment in plasma demonstrated the same regimen exceeded 90% PTA up to MIC of 4 mg/L. Sulbactam 3 g q8h 4h infusion provided PTA >90% for MIC 8 mg/L (sulbactam-intermediate), similar to previous assessment in plasma.

**Conclusion:**

Based on the ELF assessment, the maximum FDA approved dose of sulbactam (1 g q6h 0.5h infusion) provided >90% PTA for isolates with sulbactam MIC only up to 2 mg/L. Nevertheless, sulbactam 3 g q8h for 4 hours of infusion achieved higher PTA and conferred additional benefit against sulbactam-susceptible/intermediate isolates.

## Introduction

Ampicillin-sulbactam is a long-standing agent for the management of *A. baumannii* infections.^1–4^ Against this pathogen, sulbactam is the active moiety with no synergy with ampicillin.^5–7^ With the rise of antibacterial resistance, there has been an increased utilization of other antibacterial agents such as cefiderocol and the recently-approved sulbactam-durlobactam; however, ampicillin-sulbactam remains a first line therapy for infections due to susceptible *A. baumannii* and its use in clinical practice is crucial.

Despite decades worth of clinical use, sulbactam dosing has not been standardized. Per the ampicillin-sulbactam package insert, the maximum approved dose of sulbactam is 1 g q6h as 0.5 hour of infusion.^8^ The drug, however, is routinely administered at doses much larger than the approved dose. In fact, the Infectious Diseases Society of America (IDSA) recommends a dose of sulbactam 3 g q8h as a 4-hour infusion for the treatment of carbapenem-resistant *A. baumannii* infections.^9^

In addition to the lack of consensus around the dose, there is ambiguity around the susceptibility breakpoint. CLSI has published breakpoints; however, the interpretative standards were established in 1984 with the recommendation for re-evaluation when sufficient data become available.^10^ On the other hand, EUCAST has no published breakpoints.^11^ The poorly defined breakpoints could explain, at least in part, the weak correlation between MIC and clinical outcome.^12^

PK/PD analyses are critical for antimicrobial dose optimization.^13^ For the pneumonia indication, the neutropenic murine pneumonia model has been the gold standard to establish exposure targets. However, most of those preclinical investigations routinely assess the relationship between efficacy and plasma or serum concentrations.^13^ Critical, but not as readily available, are the relationships between efficacy and exposure at the target site i.e. epithelial lining fluid (ELF).^14–16^ Penetration into the ELF is an important covariate and the differences in penetration between mice and human could have implications leading to clinical dose selection failure if not accounted for during preclinical assessements.^17,18^ For example, ceftobiprole, a cephalosporin antibiotic, which demonstrated 4.5-fold less penetration into the ELF in human relative to mice, leading to drug failure at clinical development stages.^17,19^ Moreover, there are limited data directly comparing the *in vivo* efficacy of antibiotics using plasma and ELF exposures against the same clinical isolates.

We previously reported sulbactam PK/PD analysis against *A. baumannii* in the neutropenic murine pneumonia model and established plasma exposure targets necessary to achieve different magnitudes of kill.^20^ Herein, we further assessed sulbactam efficacy targets in the ELF in the same murine model and using the same clinical bacterial strains. We additionally estimated the probability of attaining the established ELF targets following clinically utilized sulbactam regimens and compared the results to those previously reported for plasma targets. Collectively, these data may serve as decision support for reassessment of the current sulbactam breakpoints.

## Methods

### Ethics

This study was approved by the Institutional Animal Care and Use Committee of Hartford Hospital. All animal experiments were conducted in concordance with the standards set by the National Research Council of the National Academy of Sciences.

### Antimicrobial agents

Commercially available ampicillin-sulbactam vials (1.5 g, Meitheal Pharmaceuticals, Chicago, IL, 60631, USA, lot ASJ008 and 1.5 g, Pfizer, New York, NY, 10017, USA, lot 33006314) were reconstituted as per the manufacturer’s directions.^8^ Dilutions were made to reach concentrations necessary to deliver mean weight-based dosing to the study mice. Treatment was administered subcutaneously as 0.1 mL injections.

### Bacterial isolates

Twenty-one *A. baumannii* isolates were obtained from the International Health Management Associates (Schaumburg, IL, USA). Isolates were collected from different geographical locations globally in the years 2018–2021. The detailed genotypic and phenotypic profiles of those isolates were previously reported.^20,21^ Isolates were of varying susceptibilities to sulbactam (MIC 1–32 mg/L) and to meropenem (MIC 0.125– >64 mg/L) as shown in Table 1 and harboured a variety of mechanisms of resistance to sulbactam and meropenem. The MICs were determined in triplicate using broth microdilution method as outlined by the CLSI.^22,23^

**Table 1.** MICs of the 21 *A. baumannii* isolates examined in the dose-ranging studies

Isolate	Country/year of collection	MIC (mg/L)-S/I/R^a^	
		Sulbactam^b^	Meropenem
ACB 280	Morocco/2020	32-R	64-R
ACB 285	France/2020	32R	64-R
ACB 309	USA/2020	32R	1-S
ACB 279	Croatia/2019	16-R	32-R
ACB 286	Spain/2020	16-R	32-R
ACB 310	USA/2019	16-R	1-S
ACB 311	Morocco/2019	16-R	1-S
ACB 287	Poland/2019	8-I	64-R
ACB 274	Israel/2021	8-I	32-R
ACB 301	France/2021	8-I	2-S
ACB 300	USA/2018	8-I	0.12-S
ACB 270	USA/2019	4-S	>64-R
ACB 272	Panama/2018	4-S	>64-R
ACB 303	USA/2019	4-S	1-S
ACB 328	Philippines/2020	2-S	32-R
ACB 332	India/2020	2-S	32-R
ACB 304	USA/2019	2-S	1-S
ACB 298	Hungary/2019	2-S	2-S
ACB 290	France/2019	1-S	0.25-S
ACB 288	Canada/2019	1-S	0.25-S
ACB 271	Germany/2019	4-S	16-R

Table 1 is a subset of Table 1 published in Abouelhassan *et al*.^21^

^a^S, susceptible; I, intermediate; R, resistant.

^b^Sulbactam concentration when MIC was tested in conjunction with ampicillin in a 2:1 ampicillin/sulbactam ratio.

### Murine neutropenic pneumonia model

Specific pathogen-free, female ICR (CD-1) mice weighing ∼20–22 g (Charles River Laboratories, Inc., MA, USA) were housed in HEPA-filtered cages in groups of six at controlled room temperature. Nourishment and enrichment were provided. Mice were administered cyclophosphamide and uranyl nitrate as previously described.^24^

Before mice lung inoculation, two transfers of the organisms (previously frozen at −80°C in skim milk) were performed onto Trypticase Soy Agar plates with 5% sheep blood (TSA II^™^; Becton, Dickinson & Co.; Sparks, MD, USA) and incubated at 37°C for ∼24 h. A bacterial suspension of 10^7^ cfu/mL in normal saline with 3% mucin was made for inoculation. Inoculum bacterial densities were confirmed by serial dilution and culture of an aliquot. Mice were anaesthetized and pneumonia was induced via intranasal inoculation with 2 hours before antimicrobial therapy initiation as previously reported.^24^

### Sulbactam bronchopulmonary PK studies

Mice were prepared as described before. Two hours after bacterial inoculation, groups of 36 mice each received different doses of ampicillin-sulbactam subcutaneously (sulbactam 1, 10, 25, 100 and 200 mg/kg). Sulbactam was dosed in combination with ampicillin using commercially available ampicillin-sulbactam vials for clinical relevancy. At specific time points, groups of six mice were euthanized by CO_2_ exposure followed by blood and bronchoalveolar (BAL) fluid collection at 4–6 time points as previously reported.^24^ Blood from each mouse was collected separately in K_2_EDTA vials for urea analysis. The blood was centrifuged and the plasma was stored at −80°C until analysis. The collected BAL fluid was centrifuged to remove any cells and was stored at −80°C until analysis. The concentrations of sulbactam and urea in the BAL fluid and urea concentrations in plasma were used to determine ELF concentrations at each of the time points; ELF sulbactam concentrations = sulbactam concentration in BAL × (plasma urea concentrations/BAL urea concentrations). The PK parameters for each of the administered doses in ELF were calculated with first-order elimination, by nonlinear least-square techniques (Phoenix WinNonlin, version 8.3, CERTARA, Princeton, NJ, USA). Compartment model selection was based on visual inspection of the fit and examination of the model diagnostics. Sulbactam penetration ratio into the ELF was estimated via simulation to predict the AUC_0-8_ and calculated as AUC_ELF(0-8)_/*f*AUC_plasma(0-8)_.

### Determination of sulbactam and urea concentration

The concentrations of sulbactam and urea were determined by a validated UPLC-MS/MS assay operated using electrospray ionization in negative ion mode using Waters ACQUITY PREMIER UPLC in tandem with a Waters Xevo TQ-XS mass spectrometer. The analytical column used in determining sulbactam and urea concentration was an ACQUITY UPLC BEH C18 1.7 µm; 2.1 × 50 mm (Waters; Milford, MA, USA; part number 186002350) and ACQUITY UPLC HSS T3 1.8 µm; 2.1 × 100 mm (Waters; Milford, MA, USA; part number 186003539), respectively.

For determining sulbactam concentrations in the BAL, a 3-minute gradient method was employed utilizing 0.1% ammonium hydroxide in water and methanol. The method used protein precipitation (methanol as solvent) to isolate sulbactam and its internal standard, sulbactam-d5 (Toronto Research Chemicals, item number S699187, lot no. 8-YSS-139-1). A calibration curve for sulbactam was prepared in saline; concentration range of 0.002–25 mg/L. The *R*^2^ value of all calibration curves analysed throughout this study was 0.99. Low- (0.05 mg/L) and high- (10 mg/L) quality control (QC) samples were prepared by a second person and evaluated throughout the study for intraday and interday accuracy and precision. Intraday average concentrations [average bias, coefficient of variance (CV)] for low- and high-QC were 0.005 mg/L (1.73%, 4.40%) and 10.01 mg/L (1.17%, 2.49%), respectively. Interday average concentrations (average bias, CV) for low- and high-QC were 0.006 mg/L (−9.19%, 2.62%) and 10.38 mg/L (3.76%, 2.80%), respectively.

For urea determination in mouse BAL, a 3-minute gradient method was employed using 0.1% formic acid in water and methanol. Both BAL fluid and plasma matrix, the latter diluted 1:20 in saline, were assayed using saline standard curves in the concentration range of 1–500 mg/L. Urea-13C (Sigma Aldrich, item number 299359; lot no. MBBB6080V) was used as the internal standard and was prepared at a concentration of 200 mg/L in water. Low- (5 mg/L), mid- (80 mg/L) and high- (400 mg/L) QC samples were prepared. Intraday average concentrations (average bias, CV) for low-, mid- and high-QC was 4.88 mg/L (−2.49%, 6.22%), 79.98 mg/L (−0.03%, 6.19%), and 408.15 mg/L (2.03%, 3.90%), respectively. Interday average concentrations (average bias, CV) for low-, mid- and high-QC were 4.92 mg/L (−1.54%, 7.02%), 80.22 mg/L (0.29%, 6.10%) and 401.47 mg/L (0.37%, 4.18%), respectively.

### Pharmacokinetics/pharmacodynamics analyses

Previously reported *in vivo* dose-ranging data in the same model against the 21 clinical *A. baumannii* strains were utilized for this analysis.^20^ Sulbactam exposures (%*f*T > MIC) in dose-ranging studies were determined through simulations using the best-fit PK parameters estimated from the single-dose PK studies. The PK parameters for the four doses of 1, 10, 25 and 100 mg/kg across the linear range were averaged to determine sulbactam exposures for doses ≤100 mg/kg in the dose-ranging studies, whereas exposures for dose of 200 mg/kg were determined through simulations using the best-fit PK parameters to the 200 mg/kg single-dose PK profile.

Using Phoenix WinNonlin, an Emax model using the Hill equation was fitted to each respective exposure versus change in log_10_ cfu/lungs at 24 h. This analysis was completed to elucidate sulbactam exposures required for stasis, 1-log kill and 2-log kill individually for the 21 isolates from the dose-ranging studies. An Emax model was also fitted to the composite curve of the 21 isolates separately.

### Population pharmacokinetics modelling in healthy volunteers

Reported mean sulbactam plasma and ELF concentrations at 1, 2.5, 3.25, 4 and 6 hours were obtained from 30 healthy volunteers administered sulbactam 1 g IV q6h as a 3-hour infusion^25^ and co-modelled using the nonparametric adaptive grid (NPAG) with adaptive gamma algorithm in the Pmetrics package for R (Laboratory of Applied Pharmacokinetics and Bioinformatics, Los Angeles, CA, USA).^26^ One- and two-compartment models were evaluated for plasma concentrations first. Final model selection was based on the goodness-of-fit by visual inspection of observed versus predicted concentration plots and Akaike information criterion (AIC).^26^ After the model was constructed in plasma, the ELF concentrations were added. No covariates were assessed due to using a single data set of the mean concentrations in plasma and ELF from a homogenous healthy volunteer population.

### Monte Carlo simulation and PTA estimation

The final population pharmacokinetic model was used as the mean parameter estimates for a 5000-patient Monte Carlo simulation in Pmetrics. The CV was artificially inflated to 40% for all parameters to be consistent with CVs observed in patients. Sulbactam dosing regimens simulated for this population included: 1 g q6h 0.5-h infusion, 2 g q12h 0.5-h infusion, 1 g q4h 0.5-h infusion, 2 g q6h 0.5-h infusion and 3 g q8h 4-h infusion.

Sulbactam PTA was determined based on the derived sulbactam PD targets in the murine neutropenic pneumonia model. The cumulative fraction of response (CFR) was calculated as the proportion of %PTA at each MIC according to the MIC distribution. Sulbactam MIC distribution was identified from a contemporary surveillance study.^27^ The percent of imipenem resistance at each MIC was used to define carbapenem resistance. Regimens that achieved PTA and CFR of at least 90% were *a priori* considered optimal.

## Results

### Sulbactam bronchopulmonary PKs

Sulbactam was detected in mouse BAL fluid following subcutaneous administration in all PK studies. ELF concentrations were best described by a one-compartment model with first-order elimination. PK parameters obtained following sulbactam administration are presented in Table 2. The resulting AUC were dose-proportional across the doses of 1–100 mg/kg in the ELF (*R*^2^ = 0.997); while deviation from proportionality was observed for the dose of 200 mg/kg. Penetration into the ELF was calculated relative to the previously reported *f*AUC_0-8_ in plasma.^20^ Sulbactam penetrated the ELF of immunocompromised mice infected with *A. baumannii* pneumonia with an ELF at free plasma ratios across doses of 1, 10, 25, 100 and 200 mg/kg of 0.66 ± 0.05. This penetration ratio was relatively consistent among the test doses and was not concentration dependent (Table 2).

**Table 2.** List of sulbactam PK parameters in plasma and ELF following single-dose sulbactam treatment, protein binding and ELF penetration

Parameter	1 mg/kg	10 mg/kg	25 mg/kg	100 mg/kg	200 mg/kg
*V*_c_/F (L/kg)	0.59	0.52	0.45	0.49	0.57
*K*_a_ (1/h)	8.28	26.71	26.79	8.64	78.44
*K*_el_ (1/h)	0.62	0.48	0.68	0.52	0.24
AUC_0-8_ (h.mg/L)^a^	2.72	38.95	79.22	385.20	1247.02
ELF penetration ratio^b^	0.58	0.67	0.69	0.70	0.67

^a^ *f*AUC_0-8_ calculated using simulated concentration based on the PK parameters.

^b^ELF penetration calculated as the ratio of AUC_0-8_ in ELF to previously reported *f*AUC_0-8_ plasma.^20^

### Pharmacokinetics/pharmacodynamics analyses

**Figure 1.**
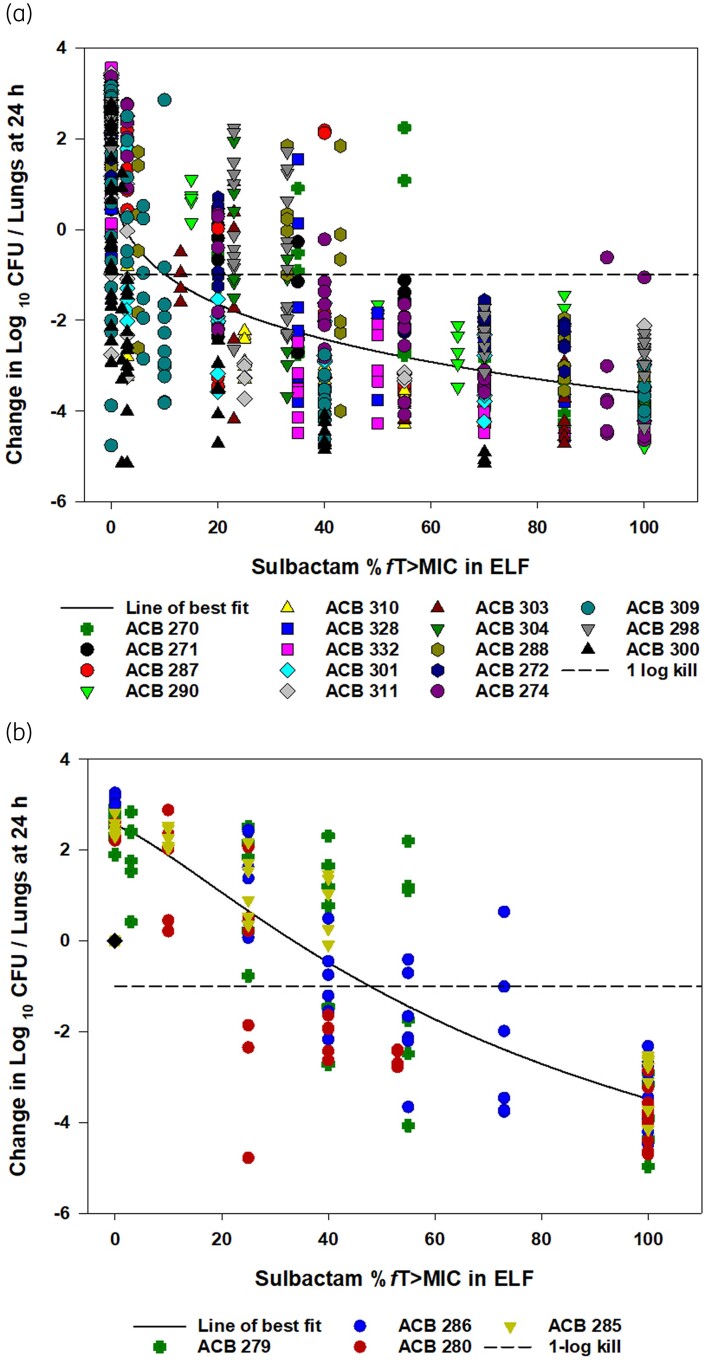
The curves of best fit to the sulbactam %*f*T > MIC in ELF and the changes in log_10_ cfu/lungs at 24 h relative to 0 h groups for the composites of examined isolates in the neutropenic pneumonia model against isolates that are susceptible or intermediate to sulbactam and isolates that are resistant to sulbactam and susceptible to meropenem (a) (*n* = 17) and isolates that are resistant to both sulbactam and meropenem (b) (*n* = 4). The solid lines represent the curves of best fit, while the symbols represent the actual changes in bacterial burdens observed in the individual mice.

PK/PD analysis in all 21 test isolates demonstrated median (IQR) ELF %*f*T > MIC targets for stasis, 1-log and 2-log kill of 8.95 (3.5–21.7), 19.94 (6.3–27.3) and 36.24 (14.9–54.9), respectively. However, similar to a previously reported PK/PD analysis in plasma for those isolates, the targets displayed a bimodal distribution.^20^ A subset of isolates that were resistant to both sulbactam and meropenem (ACB279, ACB286, ACB280 and ACB285) required much higher exposures relative to the rest of the isolate population that were not simultaneously resistant to both drugs (Figure 1). With this clear distinction in the PK/PD target among isolates, the ELF PK/PD targets were stratified into two phenotypic categories: (i) isolates that were susceptible and intermediate to sulbactam irrespective of meropenem susceptibility and isolates that were resistant to sulbactam but meropenem susceptible, and (ii) isolates resistant to both sulbactam and meropenem. Median ELF %*f*T > MIC targets required to achieve stasis, 1-log and 2-log kill by each phenotype are presented in Table 3.

**Table 3.** Sulbactam %fT > MIC plasma and ELF targets for stasis, 1-log and 2-log kill stratified according to sulbactam and meropenem susceptibilities

Number *n*	Isolate susceptibility to			Sulbactam %*f*T > MIC in ELF			
	sulbactam	Meropenem		stasis	1-log kill	2-log kill	*R* ^2^
17	S/I (and R for meropenem-S isolates only)	S/I/R	Mean	9.27	16.27	30.68	0.81
			Median	8.5	16.62	30.23	0.84
			IQR	2.78–13.2	5.3–22.0	12.0–47.3	0.7–0.9
			Composite	2.70	10.21	27.85	0.70
4	R	R	Mean	35.89	46.44	59.83	0.86
			Median	38.03	47.51	60.32	0.88
			IQR	30.7–43.2	39.7–54.2	52.8–67.4	0.8–0.9
			Composite	33.46	47.81	65.06	0.78

Data presented in mean, median with IQR and composite of the 17 and 4 isolates.

### Population pharmacokinetics modelling in healthy volunteers

Previously published sulbactam plasma and ELF concentration in healthy volunteers were utilized.^23^ The mean sulbactam plasma profile was best described by a two-compartment model (Figure S1, available as Supplementary data at *JAC-AMR* Online). The reduction in the AIC in the two-compartment model relative to the one-compartment model was by 10.5 points (AIC of 54.6 and 44.1 in the one- and two-compartment models, respectively). The ELF concentrations were then added to the existing two-compartment model to construct a three-compartment model where the ELF was the third compartment. The final parameters used as mean estimates for the Monte Carlo simulation were as follows: clearance (CL), 13.69 L/h; volume of central compartment (*V*_c_), 2.49 L; intercompartmental transfer rate constants *K*_12_, 10.65 h^−1^; *K*_21_, 21.80 h^−1^; *K*_13_, 7.47 h^−1^; *K*_31_, 1.42 h^−1^ and volume of the ELF compartment (*V*_ELF_), 2.44 L.

### Sulbactam probability of target attainment

**Figure 2.**
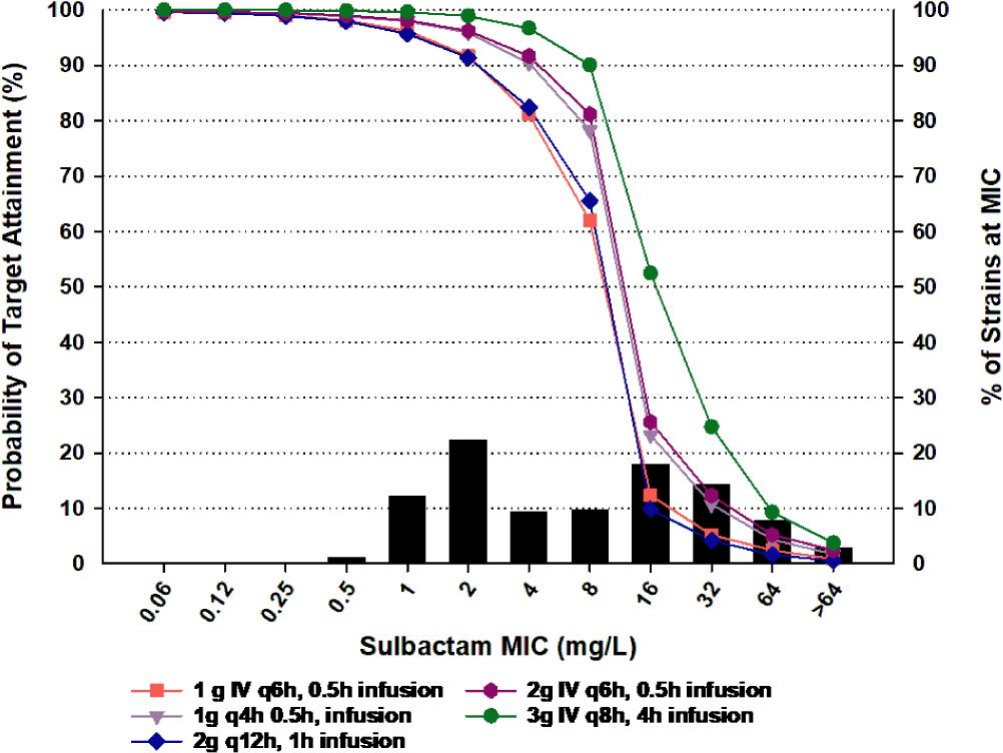
MIC distribution of sulbactam against 5032 *A. baumannii* isolates (bar graph) and the probability of target attainment for various clinically used sulbactam regimens necessary to achieve 1-log kill against *A. baumannii.*

The results of sulbactam PTA in ELF following administration of clinically utilized sulbactam regimens are reported in Table 4 and Figure 2. The targets for the PTA were stratified according to sulbactam and carbapenem phenotypes described previously. The 1-log target was used as the primary endpoint for PTA assessment because this endpoint is associated with clinical success in human.^28^ Isolates resistant to sulbactam but meropenem susceptible were not included in the PTA analysis due to their negligible distribution (<2%).^27^ Sulbactam 1 g q6h as an 0.5-h infusion provided adequate ELF exposures (PTA >90%) for MICs up to 2 mg/L. Higher doses and more frequent administration of sulbactam provide higher PTA. The 3 g q8h as a 4-h infusion regimen provided PTA exceeding 90% up to MIC of 8 mg/L (intermediate per CLSI breakpoints).

**Table 4.** The probability of target attainment (PTA %) and the cumulative fraction of response (CFR %) in ELF for various clinically used sulbactam regimens necessary to achieve 1-log kill and 2-log kill against *A. baumannii*

Sulbactam MIC (mg/L)	1 g q6h (0.5-h infusion)		2 g q12h (0.5-h infusion)		1 g q4h (0.5-h infusion)		2 g q6h (0.5-h infusion)		3 g q8h (4-h infusion)	
	PTA (%) for isolates susceptible and intermediate to sulbactam									
	1-log kill 17%*f*T > MIC	2-log kill 30%*f*T > MIC	1-log kill 17%*f*T > MIC	2-log kill 30%*f*T > MIC	1-log kill 17%*f*T > MIC	2-log kill 30%*f*T > MIC	1-log kill 17%*f*T > MIC	2-log kill 30%*f*T > MIC	1-log kill 17%*f*T > MIC	2-log kill 30%*f*T > MIC
0.0625	100	99	100	98	100	100	100	100	100	100
0.125	100	99	99	96	100	99	100	99	100	100
0.25	99	97	99	94	99	99	100	99	100	100
0.5	98	95	98	90	99	98	99	97	100	100
1	96	89	96	83	98	95	98	95	100	100
2	92	79	91	72	96	90	96	89	99	99
4	81	62	82	57	90	78	92	79	97	97
8	62	39	66	38	78	57	81	62	90	89

	PTA (%) for isolates resistant to both sulbactam and meropenem									
	1-log kill 48%*f*T > MIC	2-log kill 60%*f*T > MIC	1-log kill 48%*f*T > MIC	2-log kill 60%*f*T > MIC	1-log kill 48%*f*T > MIC	2-log kill 60%*f*T > MIC	1-log kill 48%*f*T > MIC	2-log kill 60%*f*T > MIC	1-log kill 48%*f*T > MIC	2-log kill 60%*f*T > MIC
16	12	9	10	7	23	19	26	20	52	34
32	5	4	4	3	11	8	12	9	25	16
64	2	2	2	1	4	4	5	4	9	7
>64	1	1	1	0	2	1	2	2	4	3
**CFR (%)**	**51.3**	**42.9**	**51**	**39.4**	**57.6**	**51.6**	**58.8**	**52.2**	**67.2**	**62.7**

Targets for isolates resistant to sulbactam and meropenem were separated from the rest of isolates with other phenotypic profiles.

## Discussion

PK/PD analysis for most antibiotics have been typically based on relationships established from plasma or serum concentrations. As drugs move to clinical phases, human ELF PK data become available; however, caution should be applied if assuming similar exposure magnitudes would be required in ELF to those in plasma.^29^ Recently, the scientific community and drug approval agencies have been advocating for establishing PK/PD kill targets in the ELF for the pneumonia indication.

In the present study, we performed a comprehensive PK/PD assessment of sulbactam against 21 *A. baumannii* isolates in the neutropenic murine pneumonia model. Previously, we reported sulbactam plasma PK/PD targets in the same model.^20^ As a time-dependent antibiotic, sulbactam %*f*T > MIC targets were established. Those targets were stratified based on the isolates phenotypic profile to sulbactam and meropenem. The ELF targets in the current study were similarly divided into two categories based on their phenotypic profiles. Isolates resistant to both sulbactam and meropenem required three times the exposure for the same effect compared with isolates displaying other phenotypes including those resistant to sulbactam but susceptible to meropenem. The four isolates in this study that were sulbactam-resistant/meropenem-resistant displayed a common genomic background; presence of *bla*_OXA-23_. Thus, the elevated exposure targets required for efficacy against this isolate subset were consistent with our previous reporting of lack of sulbactam *in vivo* efficacy against OXA-23-producing *A. baumannii* pneumonia using human-simulated exposures of sulbactam 1 g q6h 0.5-h infusion and 3 g q8h 4-h infusion.^21^

Overall, the ELF exposures required were generally lower than those in plasma. For example, the median 1-log kill target for sulbactam-resistant/meropenem-resistant isolates was 47.51%*f*T > MIC in the ELF versus 60.37% *f*T > MIC in plasma. This difference aligns with 0.6–0.7 sulbactam penetration into the ELF of infected mice. Notably, once the required exposure at the site of infection is established, this target can be applied during human pharmacokinetic studies of the same infection compartment to determine optimal dosing regimens.^30^

Sulbactam penetration into the pulmonary ELF of healthy and infected humans has been previously reported.^2,25^ In patients with respiratory infections, sulbactam penetrated into the alveolar lining fluid of the lung with a ratio of 0.61:1 relative to plasma.^2^ The limitations of this estimate include its evaluation a single 60-minute time point, instead of across the dosing interval, as well as referencing the penetration to total plasma concentration instead of unbound. In the study assessing healthy volunteers that was used for the Monte Carlo simulation here, the mean sulbactam ELF AUC to free plasma AUC was 0.81.^25^ Overall, the 0.66 penetration determined in this study in the murine model was largely similar to those reported in human, suggesting targets from both ELF as well as from plasma may be applicable provided penetration in infected patients is similar to those of the healthy volunteers, as has been the case with other specific beta-lactams.^31–34^ However, to account for the increased variability of drug penetration into the ELF of patients, the dispersion around the mean for all sulbactam parameter estimates was inflated to 40%CV during simulation to be consistent with potential variability reported previously in patients.^35^

The PTA based on the ELF targets compared with previously reported PTA based on plasma targets^20^ demonstrated that PTA exceeding 90% was generally one dilution of an MIC lower in the ELF. For example, the PTA in plasma of 1 g q6h, 0.5-h infusion regimen exceeded 90% up to MIC of 4 mg/L.^20^ By contrast, ELF PTA for the same regimen exceeded 90% PTA up to MIC of 2 mg/L only.

The high dose extended infusion regimen provided greater PTA than the standard dose at MICs currently defined as intermediate to sulbactam (MIC of 8 mg/L); these are similar observations to PTAs reported in plasma and adds additional clinical justification for this regimen empirically before the sulbactam MIC is known. Given that isolates resistant to both sulbactam and meropenem required higher exposures to achieve 1-log of kill relative to other phenotypes, even the high dose, extended infusion regimen did not provide PTA >90% against those isolates. To note, we opted to select the high kill target for all sulbactam-resistant isolates and not classify them according to carbapenem susceptibility because the distribution of sulbactam-resistant/carbapenem-susceptible isolates is negligible (<2%) compared with sulbactam-resistant/carbapenem-resistant isolates.^27^ Overall, despite the higher exposures with the 3-g regimen relative to the 1-g regimen, it did not achieve CFR of 90%. This was no surprise due to the global high resistance of *A. baumannii* isolates and the high exposure targets necessary to achieve kill in this population.^27^

Current CLSI sulbactam breakpoints against *A. baumannii* identify isolates with sulbactam MIC ≤ 4 mg/L as susceptible, 8 mg/L as intermediate and ≥16 mg/L as resistant.^22^ Those breakpoints align with PTA estimation following sulbactam standard dose of 1 g q6h 0.5-h infusion in plasma while the ELF PTA is suggestive of lowering the breakpoint by one dilution. However, it is important to acknowledge the limitations of utilizing healthy volunteers’ data. With the absence of infected patients sulbactam ELF concentrations, it is difficult to make conclusions on the appropriateness of the breakpoints but perhaps a more conservative approach would be to lower the breakpoint by one dilution or alternatively use a prolonged infusion high dose sulbactam regimen such as the 3 g q8h 4-h infusion for isolates with MICs = 4 mg/L.

Our study is not without limitations. Given the scarcity of data reported on the clinical ELF exposures, a few assumptions were made during our data analysis, such as estimation of the PTA in ELF by incorporating healthy volunteers’ data. The drug penetration into the ELF in healthy volunteers may be different compared with bronchopulmonary exposures among pneumonia patients.^36^ Second, because no individual data were reported for sulbactam ELF concentrations, only the mean data were fit to a compartmental model and variability was artificially inflated to 40%. However, these methods have been used in Monte Carlo simulation for many antibiotics when only healthy volunteer data are available.^37^ Finally, we simulated a sulbactam 3-g dosing regimen based on data from participants receiving 1-g dose assuming PK proportionality in human ELF exposures. Nevertheless, this study serves as the first to assess sulbactam PTA in ELF to determine the appropriateness of sulbactam clinical regimens and the findings from our analysis were in general agreement with those previously reported based on the plasma exposure analysis.

In conclusion, the sulbactam %*f*T > MIC targets in the ELF necessary to achieve various magnitude of kill against *A. baumannii* in the neutropenic murine pneumonia model was lower than identified in plasma, but was consistent with the penetration into pulmonary ELF. Using these ELF targets, a standard dosing regimen of sulbactam 1 g q6h 0.5-h infusion provides optimal coverage up to MIC of 2 mg/L whereas the IDSA-recommended dose of 3 g q8h, 4-h infusion provides additional coverage for sulbactam isolates with MICs up to 8 mg/L. Against sulbactam-resistant isolates, no clinically administered sulbactam regimen is expected to provide coverage.

## Supplementary Material

dlae203_Supplementary_Data

## Supplementary data

Figure S1 is available as Supplementary data at *JAC-AMR* Online.
