# Supplementary material for: Pharmacokinetic/pharmacodynamic analysis of sulbactam against Acinetobacter baumannii pneumonia: establishing in vivo efficacy targets in the epithelial lining fluid
Source: JAC Antimicrob Resist. 2024 Dec 20;6(6):dlae203. doi: 10.1093/jacamr/dlae203 (PMC11660682; doi:10.1093/jacamr/dlae203)

**Pharmacokinetic/ Pharmacodynamic Analysis of Sulbactam against *Acinetobacter baumannii* Pneumonia: Establishing *In Vivo* Efficacy Targets in the Epithelial Lining Fluid**

Yasmeen ABOUELHASSAN^1^, Joseph L. KUTI^1^, David P. NICOLAU^1,2^, Kamilia ABDELRAOUF^1*^

^1^ Center for Anti-Infective Research and Development, Hartford Hospital, Hartford, CT;

^2^Division of Infectious Diseases, Hartford Hospital, Hartford, Connecticut, USA

**Supplementary Figure S1:** Population pharmacokinetic model for sulbactam concentrations in a) plasma and b) ELF in healthy volunteers.


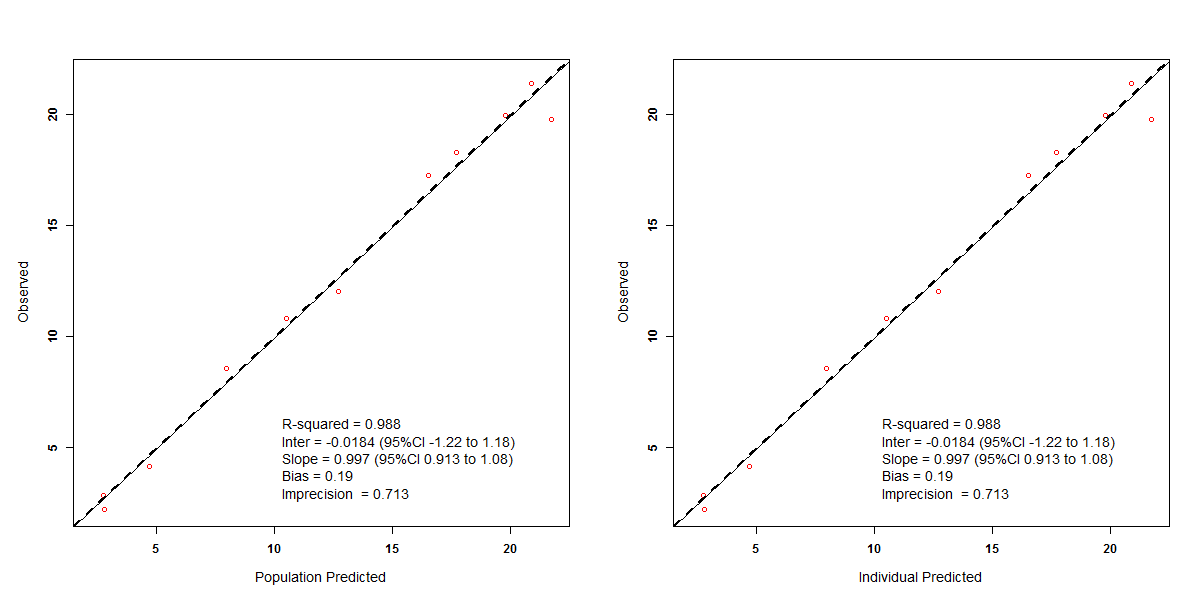


b)

a)


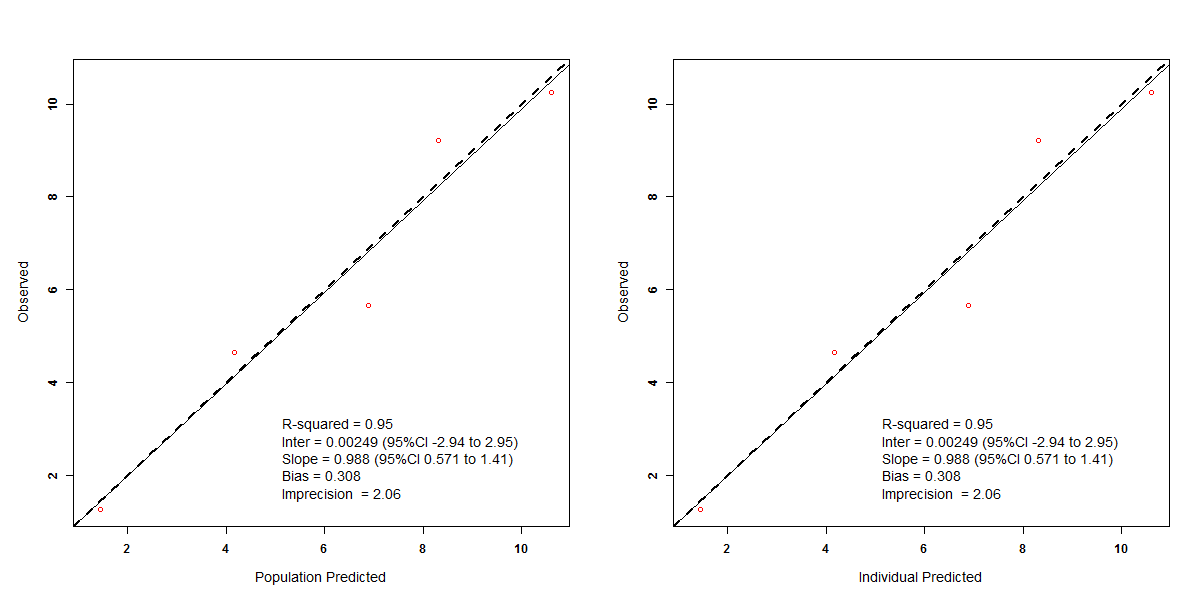

Supplement: dlae203_Supplementary_Data [file dlae203_supplementary_data.docx]
